# Frequent promoter hypermethylation of RASSF1A and CASP8 in neuroblastoma

**DOI:** 10.1186/1471-2407-6-254

**Published:** 2006-10-25

**Authors:** Paula Lázcoz, Jorge Muñoz, Manuel Nistal, Ángel Pestaña, Ignacio Encío, Javier S Castresana

**Affiliations:** 1 Departamento de Ciencias de la Salud, Universidad Pública de Navarra, Pamplona, Spain; 2 Laboratorio de Neuro-Oncología Molecular, Universidad de Navarra, Pamplona, Spain; 3 Departamento de Anatomía Patológica, Hospital La Paz, Madrid, Spain; 4 Instituto de Investigaciones Biomédicas "Alberto Sols", CSIC-UAM, Madrid, Spain; 5 Unidad de Biología de Tumores Cerebrales, Universidad de Navarra, Irunlarrea 1, E-31008 Pamplona, Spain

## Abstract

**Background:**

Epigenetic alterations and loss of heterozygosity are mechanisms of tumor suppressor gene inactivation. A new carcinogenic pathway, targeting the RAS effectors has recently been documented. RASSF1A, on 3p21.3, and NORE1A, on 1q32.1, are among the most important, representative RAS effectors.

**Methods:**

We screened the 3p21 locus for the loss of heterozygosity and the hypermethylation status of RASSF1A, NORE1A and BLU (the latter located at 3p21.3) in 41 neuroblastic tumors. The statistical relationship of these data was correlated with CASP8 hypermethylation. The expression levels of these genes, in cell lines, were analyzed by RT-PCR.

**Results:**

Loss of heterozygosity and microsatellite instability at 3p21 were detected in 14% of the analyzed tumors. Methylation was different for tumors and cell lines (tumors: 83% in RASSF1A, 3% in NORE1A, 8% in BLU and 60% in CASP8; cell lines: 100% in RASSF1A, 50% in NORE1A, 66% in BLU and 92% in CASP8). In cell lines, a correlation with lack of expression was evident for RASSF1A, but less clear for NORE1A, BLU and CASP8. We could only demonstrate a statistically significant association between hypermethylation of RASSF1A and hypermethylation of CASP8, while no association with MYCN amplification, 1p deletion, and/or aggressive histological pattern of the tumor was demonstrated.

**Conclusion:**

1) LOH at 3p21 appears in a small percentage of neuroblastomas, indicating that a candidate tumor suppressor gene of neuroblastic tumors is not located in this region.

2) Promoter hypermethylation of RASSF1A and CASP8 occurs at a high frequency in neuroblastomas.

## Background

Neuroblastic tumors are childhood embryonal neoplasms composed of neural crest-derived neuroectodermal cells, which migrate to the adrenal medulla and sympathetic nervous system[1]. According to the schwannian stroma component, these tumors are classified as neuroblastoma, nodular or intermixed ganglioneuroblastoma, and ganglioneuroma[1]. Neuroblastoma is the most common extracranial malignant pediatric solid tumor. The genetic alterations most frequently found in neuroblastoma are MYCN amplification and 1p36 allelic loss, both of them associated with a poor prognosis. Gains of chromosomes 4q, 6p, 7q, 11q and 18q, amplification of MDM2 and MYCL genes, 17q trisomy and allelic losses at 11q, 14q and 10q have also been described[2].

Allelic losses on the short arm of chromosome 3 have been found in many sporadic human cancers, including lung, kidney, breast, ovarian and neural crest-derived tumors[3]. Loss of heterozygosity (LOH) studies have identified several distinct 3p loci which are likely to contain the tumor suppressor genes: 3p12, 3p14, 3p21.3 and 3p25-26. Overlapping homozygous deletions in lung and breast tumor cell lines have identified the minimal deleted region to 120 kb in 3p21.3 [3-6]. Eight genes were located within this region including the alpha-2 delta-2 calcium channel subunit, PL6, 101F6, NPRL2/G21, BLU, RASSF1, FUS1 and LUCA2[5].

RASSF1A (Ras Association Domain Family Protein 1 isoform A) was described by Dammann et al[7] as a Ras effector located at 3p21.3 and its function was thought to be in modulating the Ras-mediated apoptotic response. This gene is also involved in maintaining cytoskeletal integrity[8] and regulating mitosis[9]. RASSF1A is subject to promoter hypermethylation in several neoplasias such as lung, breast, ovarian and kidney cancers and in pediatric tumors[7,10-12].

BLU shares 30–32% identity with proteins of the MTG/ETO family of transcription factors and the suppressins, which are thought to regulate cell cycle entry[3]. BLU promoter hypermethylation has been described in lung, breast, kidney, and neuroblastoma cell lines[3]. In these cases, hypermethylation was correlated with down regulation of BLU expression. A correlation between methylation of BLU and RASSF1A has been described in NSCLC[3].

Another Ras effector described recently is the NORE1[13]. NORE1, in 1q32.1, is homologous to the previously described mouse *Nore1 *gene and exists in the 3 alternatively spliced isoforms, NORE1Aα, NORE1Aβ and NORE1B[14]. NORE1 and RASSF1A efficiently form homodimers and heterodimers, with each other, through their non-homologous aminoterminal segments. The ability of RASSF1A to form heterodimers with NORE1, thereby associating with Ras-like GTPases, may be important for its function as a tumor suppressor gene[15]. NORE1A shares sequence similarity and genomic structure with RASSF1A[15]. Promoter hypermethylation of NORE1A has been described in lung, breast, colon, kidney and Wilms' tumors[14,16].

Apoptotic defects may be responsible for tumor formation, progression and resistance to drugs in neuroblastoma. The best known apoptotic defect in neuroblastoma tumors and cell lines is the down regulation of CASP8, which strongly correlates with TRAIL unresponsiveness. CASP8, located at chromosome 2q33-q34, encodes caspase 8, an initiator caspase that plays an important role in the Fas-Fas ligand pathway[17,18]. Alterations of these genes have been described in several neoplasias, such as mutations in colon cancer and promoter hypermethylation in medulloblastomas and neuroblastomas [19-21].

In this study we analyzed: 1) LOH and MSI (microsatellite instability) at chromosome 3p21 in neuroblastic tumors; 2) promoter methylation of the RASSF1A, NORE1A, BLU and CASP8 genes, determined in neuroblastic tumors and neuroblastoma cell lines; and 3) gene expression and its correlation with promoter methylation upstream of these genes, analyzed in neuroblastoma cell lines.

## Methods

### Tumor samples

Forty one neuroblastic tumors (26 neuroblastomas, 12 ganglioneuroblastomas and 3 ganglioneuromas) were used for this study (Table 1). All tumors were frozen and stored at -80°C immediately after surgical extraction. DNA isolated from blood samples of 21 patients was used as negative control for the LOH analysis. Samples were collected at Hospital La Paz, Madrid, Spain. Clinicopathological sample data included: patient age and sex, tumor location, diagnosis, Shimada's index, and cell line proliferation index (Ki67).

### Cell lines

Neuroblastoma cell lines SK-N-DZ, SK-N-SH, SK-N-Be(2), SK-N-FI, BE(2)C, and MC-IXC were purchased from the American Type Culture Collection (ATCC, Manassas, VA), and IMR-32, Kelly, SIMA, SH-SY5Y, SK-N-MC, and MHH-NB-11 were purchased from the Deutsche Sammlung von Mikroorganismen und Zellkuturen Gmbh (DSMZ, Braunchsweig, Germany).

### Nucleic acids extraction

DNA from tumor specimens was isolated by phenol-chloroform extraction. Tissues were resuspended with 1 ml lysis solution buffer (0.5 M NaCl, 50 mM Tris-HCl pH 7.6, 50 mM EDTA, and 0.5% SDS) and incubated with 20 μl proteinase K (20 mg/ml) at 56°C, overnight. Next, 0.4 ml saturated NaCl was added to the mixture, the sample shaken, and spun at 13.000 rpm for 10 min. The supernatant was transferred to a new tube, and DNA purified by phenol-chloroform extraction and precipitated by adding 2 volumes of absolute ethanol at -80°C. The DNA was washed twice with 70% ethanol, dried, and resuspended with 200–500 μl Tris-EDTA Buffer (10 mM Tris-HCl, 1 mM EDTA, pH 8).

DNA from cell lines was extracted using the Wizard Genomic DNA Purification Kit (Promega Corporation, Madison, USA), following to the manufacturer's protocol.

RNA from cell lines was extracted with the QuickPrep™ Total RNA Extraction Kit (Amersham Biosciences, Buckinghamshire, UK), following to the manufacturer's instructions.

### LOH and MSI analysis

Blood and tumor DNA from 21 patients was screened for LOH and analyzed for MSI using four polymorphic markers (D3S3507, D3S3615, D3S3624 and D3S3522) located on chromosome 3p21.

Approximately 50 ng of DNA template was amplified in a total volume of 25 μl containing 5 pmol of each primer, 2 mM MgCl_2_, 0,2 mM of each dNTP, 10× reaction buffer and 1 unit of Biotaq™ DNA polymerase (Bioline Ltd., London, UK). For D3S3522 and D3S3615 amplification reactions were first heated to 95°C for 5 min, followed by 32 cycles of 94°C (30 s), 58°C (30 s), and 72°C (30 s). A final step at 72°C for 10 min was included. The annealing temperatures used for D3S3624 and D3S3504 were 56°C and 60°C, respectively.

After amplification, 17 μl of the PCR products were analyzed by electrophoresis using 15% polyacrylamide gels. The DNA bands were visualized by ethidium bromide staining (0,5 μg/ml).

### Sodium bisulfite modification

DNA from the 41 tumors and the 12 cell lines was modified with sodium bisulfite using the CpGenome™ DNA Modification Kit (Chemicon International, Inc. Temecula, CA, USA) according to the manufacturer's instructions.

### MSP

PCRs were carried out in a final volume of 25 μl containing 2,5 mM MgCl_2_, 0,2 mM of each dNTP, 10× reaction buffer, 10 pmol of each primer, 5% of DMSO, 60 ng of modified DNA and 1 unit of AmpliTaq Gold™ DNA polymerase (Applied Biosystems, Foster City, CA, USA). Annealing temperatures were from 50°C to 66°C. Primers for RASSF1A amplification were designed by using the MethPrimer^® ^software [22], and primers for NORE1A, BLU and CASP8 had been previously described [3,14,23] (see Table 2).

### RT-PCR

One μg of RNA from each cell line was mixed with 1 μl 10 mM dNTPs, and 1 μl 250 μM random primers in a final volume of 12 μl. After heating to 65°C for 5 min, 4 μl 5× reaction buffer and 2 μl 0.1 mM DTT were added. The mixture was incubated at 42°C for 2 min, followed by addition of 1 μl of Superscript™ II RNA Retrotranscriptase (Invitrogen™ Life Technologies, Carlsbad, CA). Samples were incubated at 25° for 10 min, and then at 42°C for 50 min. A final step at 70°C for 15 min was included. cDNAs were stored at -20°C until their use.

Approximately 75 ng of cDNA was amplified in a total volume of 25 μl containing 0,2 mM of each dNTP, 2 mM MgCl_2_, 10× reaction buffer, 10 pmol of each primer, 5% of DMSO, and 1 unit of AmpliTaq Gold™ DNA polymerase (Applied Biosystems, Foster City, CA, USA). For BLU amplification, no DMSO was added and 1 unit of Biotaq™ DNA polymerase (Bioline Ltd., London, UK) was used. Primers for RASSF1A and TFR amplification were designed with the Oligo 4.0 software (NBI, Hamel, MN, USA), and the CASP8 primers were designed using the Primer Express 2.0 software. Primers used for NORE1A and BLU PCRs had been previously described[3,14] (see Table 3). A fragment of the transferrin receptor gene (TFR) was amplified as an internal control.

### Statistical analysis and ethics

Fisher's exact test was applied to screen for any statistical relationship between LOH at 3p21, promoter hypermethylation of the RASSF1A, NORE1A, BLU and CASP8 genes and the clinicopathological tumor data. Also, statistical correlation between RASSF1A and BLU hypermethylation, and between RASSF1A and CASP8 hypermethylation was analyzed. Ethical approval for the study was obtained from the Ethics Committee of the University of Navarra, in Pamplona, Spain (ref. 38/2002)

## Results

### LOH and MSI analysis

**Figure 1:**
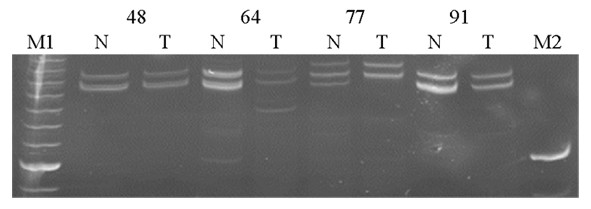
Loss of heterozygosity study at D3S3522 polymorphic marker in neuroblastic tumors. PCR products were run in a 15% polyacrylamide gel and visualized after ethidium bromide staining (0.5 μg/ml) T: DNA from tumor tissue; N: DNA from peripheral blood of the same patient; M1/M2: 10 bp DNA ladder/1 Kb Plus DNA ladder (Invitrogen™ Life and Technologies, Carlsbad, CA, USA). Sample number 64 showed microsatellite instability.

LOH and MSI were determined using four polymorphic markers (D3S3507, D3S3615, D3S3624 and D3S3522) located on chromosome 3p21. We found LOH at the markers D3S3507 and D3S3615 in 1 (6%) of 16 and 2 (14%) of 14 neuroblastic tumors, respectively. MSI was detected for all polymorphic markers studied (Figure 1, Table 4).

### Methylation

**Figure 2:**
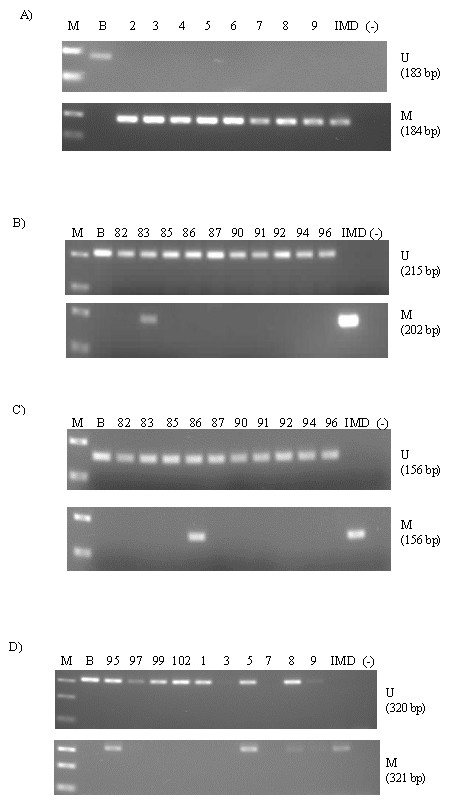
Promoter methylation analysis of RASSF1A (A), NORE1A (B), BLU (C) and CASP8 (D) in neuroblastic tumors (B, C and D) and cell lines (A), determined by MSP and visualized in 2.5% agarose gels stained with 0.1 μg/ml ethidium bromide. U/M: unmethylated/methylated; M: 1 Kb Plus DNA Ladder (Invitrogen™ Life and Technologies, Carlsbad, CA, USA); B: DNA obtained from blood of a normal donor; IMD (*in vitro methylated DNA*): positive control, CpGenome™ Universal Methylated DNA (Chemicon International, Inc., Temecula, CA, USA);-: water (no DNA negative control). A) 2: SH-SY5Y; 3: SK-N-SH; 4: SK-N-MC; 5: IMR-32; 6: MHH-NB-11; 7: SK-N-Be(2); 8: SK-N-DZ; 9: BE(2)C. B), C), and D) Numbers on top of each well correspond to DNA from different neuroblastic tumors analyzed.

RASSF1A was analyzed in 35 neuroblastic tumors and in 12 neuroblastoma cell lines. Hypermethylation of this gene was detected in 29 (83%) tumors and in all cell lines (Figure 2, Tables 4 and 5).

BLU hypermethylation was detected in 3 (8%) of 35 neuroblastic tumors, and in 7 (66%) neuroblastoma cell lines (Tables 4 and 5). We did not detect any statistical correlation between RASSF1A and BLU hypermethylation.

Only one (3%) of 34 neuroblastic tumors showed NORE1A hypermethylation, but 6 (50%) cell lines showed this alteration (Tables 4 and 5).

CASP8 promoter hypermethylation was studied in a total of 35 neuroblastic tumors and detected in 21 (60%) of these. Eleven (92%) of the 12 neuroblastoma cell lines studied showed hypermethylation of the CASP8 promoter (Tables 4 and 5). RASSF1A and CASP8 hypermethylation was linked (Fisher's exact test, p < 0.05).

### RT-PCR

**Figure 3:**
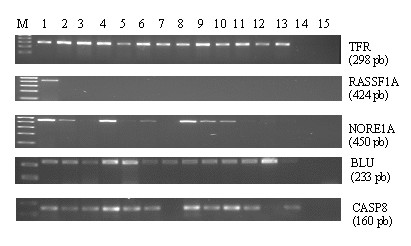
Expression of RASSF1A, NORE1A, BLU and CASP8 in neuroblastoma cell lines determined by RT-PCR and visualized in 2% agarose gels stained with 0.1 μg/ml ethidium bromide. M: 1 Kb Plus DNA Ladder (Invitrogen™ Life and Technologies, Carlsbad, CA, USA); 1: Normal human lung cDNA; 2: Kelly; 3: BE(2)-C; 4: SK-N-MC; 5: SK-N-FI; 6: SK-N-Be(2); 7: IMR-32; 8: MC-IXC; 9: SH-SY5Y; 10: SK-N-SH; 11: MHH-NB-11; 12: SIMA; 13: SK-N-DZ; 14: genomic DNA; 15: water. A fragment of the transferrin receptor gene was amplified as an internal control.

We studied the expression of the RASSF1A, BLU, NORE1A and CASP8 genes in 12 neuroblastoma cell lines by semi-quantitative RT-PCR (Figure 3, Table 5). RASSF1A showed lack of expression in all cell lines. BLU was expressed in all cell lines. NORE1A and CASP8 showed expression in all cell lines except in IMR-32. In conclusion, gene methylation was correlated with lack of expression only in the case of RASSF1A.

### Statistical analysis

Fisher's exact test did not reveal any statistical correlation between the genetic alterations found in tumor samples and the clinicopathological data.

## Discussion

Loss of heterozygosity (monoallelic loss) associated with hemimethylation (methylation at the promoter of the same gene in the remaining allele) or with mutation of the remainder allele, constitute two mechanisms of inactivation of tumor suppressor genes. A third one might be hypermethylation of the gene at both alleles.

LOH and MSI are frequent genetic alterations found in neoplasias. For example, in colorectal cancers, high frequency of LOH is associated with high metastatic potential[24]. LOH at 12q22-q34 and 14q has been described in glioblastomas, while MSI is not frequent in these tumors [25-27].

Allelic losses of chromosome 3p are frequent in many sporadic cancers, such as lung, colon, nasopharyngeal and gastrointestinal tract tumors [28-31]. The genetic alteration most frequently found in neuroblastoma is 1p36 allelic loss, which is associated with MYCN amplification and poor prognosis. LOH at other regions, like 14q, 11q, 9p and 10q, has also been described[32,33]. A recent report found a correlation between LOH at 3p21 and neuroblastoma stages 1 and 4 without MYCN amplification[34]. In our study, we analyzed LOH and MSI at chromosome 3p21, using four polymorphic markers, in 21 neuroblastic tumors, and found LOH and MSI in 14% of the tumors. Our results are similar to previously published data[34,35] that describes LOH and MSI at 3p in a small percentage of neuroblastic tumors.

Aberrant promoter methylation of tumor suppressor genes is a mechanism of inactivation described in many tumors. In neuroblastomas, methylation of several genes, including MGMT, SLIT2, CD44, FLIP and RASSF1A, has been described[3,23,36-39]. Moreover, CpG methylation profiles characterize different clinical subgroups and strongly correlate with outcome[40,41]. In our study, we examined the methylation status of the RASSF1A, BLU, NORE1A and CASP8 genes in 41 neuroblastic tumors and in 12 neuroblastoma cell lines.

Epigenetic inactivation of RASSF1A has been described in sporadic tumors such as gliomas, breast, prostate, kidney and ovarian tumors[11,12,42-44], and also in the pediatric tumors Wilms' tumor, neuroblastoma, medulloblastoma, rhabdomyosarcoma and retinoblastoma[10,45]. In neuroblastoma several studies have shown a high frequency of RASSF1A methylation. Yang *et al. *found a statistical correlation between RASSF1A methylation and poor outcome[36], while Wong *et al.*[46] did not detect any link between clinical data (age, tumor size and outcome) and methylation of this gene. Furthermore, they found RASSF1A methylation in adjacent non-tumor tissues, suggesting that this epigenetic change is potentially an early and critical event in childhood neoplasia. In our study, we found RASSF1A hypermethylation in 83% of the tumor samples. RASSF1A was the only gene for which we could see a significant correlation between promoter hypermethylation and reduction or lack of expression. All neuroblastoma cell lines analyzed showed hypermethylation of RASSF1A and none expressed the gene.

In order to determine if RASSF1A methylation was due to global methylation of the chromosomal region 3p21.3, or whether it was gene specific, we analyzed BLU promoter methylation in the neuroblastic tumor samples and neuroblastoma cell lines. Agathanggelou *et al. *reported a BLU methylation frequency of 41% in neuroblastoma tumors and of 86% in neuroblastoma cell lines[3]. We found BLU methylation in 8% of the tumor samples and in 66% of the cell lines. All cell lines analyzed expressed the RASSF1A gene. Our results suggest that methylation of RASSF1A and BLU are independent events. Agathanggelou *et al. *also found no correlation between methylation of RASSF1A and BLU in neuroblastoma and SCLC, while in NSCLC, they described a global methylation pattern of the 3p21.3 region[3].

The NORE1A gene is frequently methylated in lung, breast, colon, kidney and Wilms' tumors, while no mutation of this gene has been described in cancer[14,16]. We found NORE1A methylation in 3% of the tumor samples and in 50% of the neuroblastoma cell lines. Only the IMR-32 cell line showed lack of NORE1A expression. The difference in methylation percentage between tumors and cell lines suggests that NORE1A methylation appears later in tumor progression. An alternative explanation is that cell culture induces methylation changes[47]. To our knowledge, this is the first report to show NORE1A promoter methylation and lack of expression in neuroblastoma.

The main apoptotic defect found in neuroblastomas is the down-regulation of the CASP8 gene. Aberrant methylation of this gene has been reported in the childhood neoplasias medulloblastoma, retinoblastoma and neuroblastoma[19,48]. Several studies suggest that CASP8 promoter methylation is associated with MYCN amplification, while other reports claim that there is no correlation between these genetic alterations[21,49-51]. We found CASP8 promoter methylation in 60% of the neuroblastic tumors and in 92% of the neuroblastoma cell lines. All cell lines, except IMR-32, showed expression of the CASP8 gene. In this study, we did not find a correlation between CASP8 methylation and MYCN amplification, but we found a statistical relationship between CASP8 and RASSF1A promoter methylation, as previously described in different pediatric tumors by Harada *et al. *[48].

## Conclusion

Our results suggest that: 1) LOH at 3p21 appears in a small percentage of neuroblastomas, suggesting that a candidate tumor suppressor gene of neuroblastic tumors is not located in this region, and 2) promoter hypermethylation of RASSF1A and CASP8 is frequent in neuroblastomas.

## Abbreviations

DMSO: dimethyl sulfoxide

dNTP: deoxynucleoside triphosphate

DTT: dithiothreitol

EDTA: ethylenediaminetetraacetic acid

LOH: loss of heterozygosity

MSI: microsatellite instability

MSP: methylation specific PCR

NSCLC: non small cell lung cancer

SCLC: small cell lung cancer

TFR: transferrin receptor gene

## Competing interests

The author(s) declare that they have no competing interests.

## Authors' contributions

PL and JM carried out the molecular genetic studies under the supervision of IE and JSC. PL also participated in the study design and drafting of the manuscript. MN confirmed the pathological diagnosis of the tumor samples. AP was in charge of the neuroblastoma samples data base. JSC conceived of the study, participated in its design, coordination and in the draft of the manuscript. All authors read and approved the final manuscript.

## Pre-publication history

The pre-publication history for this paper can be accessed here:



## Figures and Tables

**Table 1:** Clinico-pathological and genetic data of 41 neuroblastic tumors.

**Tumor**	**Age**	**Sex**	**Localization**	**Diagnosis**	**Shimada Histology**	**Ki 67**	**LOH 1p**	**MYCN amp.**
1	3 y	M	Mediastinum	GN	n.a.	1%	n.a.	n.a.
17	13 y	M	Mediastinum	GN	Stroma rich. Dif. Low MKI.	1%	n.a.	n.a.
94	4 y	M	Suprarenal	GN	Stroma rich. Dif. Low MKI.	15%	n.a.	n.a.
3	5 y	M	Suprarenal	GNB	Stroma rich. Dif. Low MKI.	5%	n.a.	n.a.
5	3 y	F	Suprarenal	GNB	Stroma rich. Nodular	5%	NO	NO
7	3 y	F	Retroperitoneum	GNB	Stroma rich. Mixed	2%	NO	NO
8	1 y	F	Suprarenal	GNB	Stroma rich. Nodular	n.a.	NO	YES (10 copies)
9	7 y	M	n.a.	GNB	Stroma rich. Dif. Low MKI.	10%	NO	NO
10	4 y	M	Retroperitoneum	GNB	Stroma rich. Dif. Mild MKI.	15%	YES	NO
61	1 y	F	Mediastinum	GNB	Stroma rich. Dif. Low MKI.	10%	n.a.	NO
82	2 y	M	Retroperitoneum	GNB	Stroma rich. Nodular	n.a.	NO	NO
83	6 y	V	Mediastinum	GNB	Stroma rich. Nodular	15%	NO	NO
86	2 y	F	Retroperitoneum	GNB	Stroma rich. Dif. Low MKI.	15%	n.a.	YES (10–50 copies)
92	6 y	M	Mediastinum	GNB	Stroma rich. Nodular	15%	NO	NO
107	1 y	M	Thoracic-abdominal	GNB	Stroma rich. Mixed	5%	YES	NO
12	2 m	F	Suprarenal	NB	Stroma poor. Undif. Mild MKI.	50%	NO	YES (20 copies)
13	11 m	M	Abdominal	NB	Stroma poor. Undif. High MKI.	60%	NO	NO
14	2 y	M	Retroperitoneum	NB	Stroma poor. Dif. Low MKI.	40–50%	YES	YES (50 copies)
15	3 y	M	Suprarenal	NB	Stroma poor. Dif. Mild MKI.	40–50%	NO	NO
16	9 y	M	Mediastinum	NB	Stroma poor. Undif. High MKI.	60%	NO	NO
18	6 y	F	Thoracic-abdominal	NB	Stroma poor. Undif. Mild MKI.	60%	YES	NO
33	9 y	M	Suprarenal	NB	Stroma poor. Undif. High MKI.	70%	NO	NO
37	2 w	F	Retroperitoneum	NB	Stroma Poor. Undif. Low MKI.	10%	NO	NO
34	2 m	F	Mediastinum	NB	Stroma poor. Dif.	15%	YES	NO
45	3 w	F	Suprarenal	NB	Stroma poor. Undif. Low MKI.	10%	YES	NO
48	7 m	F	Paraaortic	NB	Stroma rich. Dif Low MKI.	5%	n.a.	NO
51	4 w	M	Suprarenal	NB	Stroma poor. Dif.	20%	NO	NO
52	1 m	F	Suprarenal	NB	Stroma poor. Undif. Low MKI.	10%	n.a.	NO
63	5 m	M	Suprarenal	NB	Stroma poor. Undif.	40%	NO	NO
64	2 y	M	Suprarenal	NB	Stroma poor. Undif.	n.a.	n.a.	NO
73	2 y	F	Suprar. retroper	NB	Stroma poor. Undif.	85%	YES	YES
77	4 y	M	Retroperitoneum	NB	Stroma poor. Undif.	25%	NO	NO
85	4 y	M	Suprarenal	NB	Stroma poor. Undif. Mild MKI.	30%	n.a.	NO
87	3 y	M	Hypochondrium	NB	Stroma poor. Undif. High MKI.	30%	n.a.	YES (10 copies)
90	12 y	n.a.	Brain	NB	n.a.	n.a.	n.a.	n.a.
91	3 y	F	Retroperitoneum	NB	Stroma poor. Undif.	25%	NO	NO
95	4 m	F	Suprarenal	NB	Stroma poor. Undif.	n.a.	YES	n.a.
96	3 y	F	n.a.	NB	Stroma poor. Undif.	50%	n.a.	n.a.
97	4 m	F	Suprarenal	NB	Stroma poor. Undif.	10%	NO	n.a.
99	2 m	F	Thoracic-abdominal	NB	Stroma poor. Undif. High MKI.	60%	n.a.	n.a.
102	1 y	F	Mediastinum	NB	Stroma poor. Undif. Low MKI.	15%	YES	NO

F: female. M: man. y: year/years. M: month/months. w: weeks. GN: Ganglioneuroma. GNB: Ganglioneuroblastoma. NB: Neuroblastoma. Dif: differentiated. Undif: undiferentiated. MKI: mitosis-karyorrhexis index. MYCN amp.: MYCN amplification. n.a.: data non avaible.

**Table 2:** Primers and conditions for MSPs.

**Gene**	**Primer forward (5'–3')**	**Primer reverse (5'–3')**	**Size (bp)**	**T (°C)**	**Cycles**
RASSF1A					
- U	GAGAGTGTGTTTAGTTTTGTTTTTG	CCCATACTTCACTAACTTTAAACAC	183	62	38
- M	GAGAGCGCGTTTAGTTTCGTTTTC	ACCCGTACTTCGCTAACTTTAAACG	184	58	35
					
NORE1A					
- U	ATTTATATTTGTGTAGATGTTGTTTGGTAT	ACTTTAACAACAACAACTTTAACAACTACA	215	54	40
- M	CGTCGTTTGGTACGGATTTTATTTTTTTCGGTTC	GACAACTTTAACAACGACGACTTTAACGACTACG	202	62	40
					
BLU					
- U	TTTGTGGGTTATAGTTTGAGAAAGTG	AACAAATTAACCACACCTACAC	156	60	30
- M	TTCGTGGGTTATAGTTCGAGAAAGCG	AACGAATTAACCGCGCCTACGC	156	66	30
					
CASP8					
- U	TAGGGGATTTGGAGATTGTGA	CCATATATCTACATTCAAAACAA	320	53	40
- M	TAGGGGATTCGGAGATTGCGA	CGTATATCTACATTCGAAACGA	321	50	40

U: unmethylated; M: methylated.

**Table 3:** Primers and conditions for RT-PCRs.

**Gene**	**Primer forward (5'–3')**	**Primer reverse (5'–3')**	**Size (bp)**	**T (°C)**	**Cycles**
RASSF1A	TCTGTGGCGACTTCATCTGG	TTGGGCAGGTAAAAGGAAGT	424	60	35
					
NORE1A	ATCCAGTTGGACTGCAGTCA	GGGATTGTCCACAACCATG	450	60	35
					
BLU	GCTTAGCACACACAACCTGC	CCTTGGCAAAACTTGTGAGG	233	67	30
					
CASP8	AGAGAAGCAGCAGCCTTGAAGG	AGACAGTATCCCCGAGGTTTGC	160	60	35
					
TFR	GTCAATGTCCCAAACGTCACCAGA	ATTTCGGGAATGCTGAGAAAACAGACAGA	298	60	30

**Table 4:** LOH/MSI and promoter methylation in 41 neuroblastic tumors.

**Tumor**	**Diagnostic**	**LOH/MSI**				**Methylation**			
		**D3S3615**	**D3S3507**	**D3S3624**	**D3S3522**	**RASSF1A**	**NORE1A**	**BLU**	**CASP8**

**1**	GN	-	-	-	-	-	-	-	-
**17**	GN	n.d.	n.d.	n.d.	n.d.	○	-	-	-
**94**	GN	n.d.	n.d.	n.d.	n.d.	○	-	-	-
**3**	GNB	n.d.	n.d.	n.d.	n.d.	○	-	-	-
**5**	GNB	n.d.	-	n.d.	n.d.	○	n.a.	○	○
**7**	GNB	n.d.	n.d.	n.d.	n.d.	n.a.	n.a.	n.a.	n.a.
**8**	GNB	MSI	MSI	MSI	MSI	○	-	-	○
**9**	GNB	n.d.	n.d.	n.d.	n.d.	○	-	-	○
**10**	GNB	n.d.	n.d.	n.d.	n.d.	●	-	-	-
**61**	GNB	n.d.	n.d.	n.d.	n.d.	-	-	-	-
**82**	GNB	n.d.	-	n.d.	n.d.	-	-	-	-
**83**	GNB	n.d.	n.d.	n.d.	n.d.	○	○	-	○
**86**	GNB	n.d.	n.d.	n.d.	n.d.	●	-	○	○
**92**	GNB	-	-	-	-	○	-	-	○
**107**	GNB	n.d.	n.d.	n.d.	n.d.	○	-	-	○
**12**	NB	n.d.	n.d.	n.d.	n.d.	○	n.a.	-	○
**13**	NB	n.d.	n.d.	n.d.	n.d.	○	-	-	○
**14**	NB	n.d.	n.d.	n.d.	n.d.	n.a.	n.a.	n.a.	n.a.
**15**	NB	n.d.	n.d.	n.d.	n.d.	n.a.	n.a.	n.a.	n.a.
**16**	NB	n.d.	n.d.	n.d.	n.d.	n.a.	n.a.	n.a.	n.a.
**18**	NB	n.d.	n.a.	n.a.	n.a.	○	-	-	-
**33**	NB	n.d.	n.d.	n.d.	n.d.	○	-	-	-
**37**	NB	n.d.	n.a.	n.a.	n.a.	n.a.	n.a.	n.a.	n.a.
**34**	NB	-	-	-	-	n.a.	n.a.	n.a.	n.a.
**45**	NB	LOH	LOH	-	-	○	-	-	○
**48**	NB	-	-	-	-	●	-	-	○
**51**	NB	n.a.	n.a.	n.a.	n.a.	○	-	-	○
**52**	NB	n.d.	n.d.	n.d.	n.d.	○	-	-	-
**63**	NB	-	-	-	-	○	-	-	○
**64**	NB	MSI	-	MSI	MSI	○	-	○	○
**73**	NB	n.a.	n.a.	n.a.	n.a.	○	-	-	○
**77**	NB	LOH	-	MSI	LOH	○	-	-	○
**85**	NB	n.d.	n.d.	n.d.	n.d.	○	-	○	-
**87**	NB	n.d.	n.d.	n.d.	n.d.	○	-	-	○
**90**	NB	-	-	-	-	-	-	-	○
**91**	NB	-	-	-	-	○	-	-	○
**95**	NB	-	-	-	-	○	-	-	○
**96**	NB	n.d.	n.d.	n.d.	n.d.	-	-	-	-
**97**	NB	-	-	-	-	○	-	-	-
**99**	NB	n.d.	n.d.	n.d.	n.d.	○	-	-	-
**102**	NB	-	-	-	-	○	-	-	-

GN: ganglioneuroma; GNB: ganglioneuroblastoma; NB: neuroblastoma; ●: Methylated; ○: Hemimethylated;-: unmethylated, no LOH/MSI; n.d.: not determined (due to lack of blood specimens from patients); n.a.: non amplified.

**Table 5:** Promoter hypermethylation and expression in neuroblastoma cell lines.

Cell line	Methylation				Expression			
		
	RASSF1A	BLU	NORE1A	CASP8	RASSF1A	BLU	NORE1A	CASP8
Kelly	●	○	○	○	-	+	-/+	+
SIMA	●	○	-	○	-	+	-/+	-/+
MC-IXC	●	-	-	○	-	+	+	+
SK-N-FI	●	-	-	○	-	+	-/+	+
SH-SY5Y	●	○	-	○	-	+	+	+
SK-N-SH	●	○	-	○	-	+	-/+	+
SK-N-MC	●	-	○	-	-	+	+	+
IMR-32	●	○	○	○	-	+	-	-
MHH-NB-11	●	○	○	○	-	+	-/+	-/+
SK-N-Be(2)	●	-	-	○	-	+	-/+	+
SK-N-DZ	●	○	○	○	-	-/+	-/+	-/+
BE(2)C	●	-	○	○	-	+	-/+	+

●: Methylated; ○: Hemimethylated;-: unmethylated/lack of expression; +: expression; -/+: low expression.
